# Measuring Abdominal Skin‐Surface Distances Using Photos for Perforator Mapping Analysis—A Validation Study on 3D‐Printed DIEP‐Flap Models

**DOI:** 10.1002/rcs.70108

**Published:** 2025-09-17

**Authors:** Fabian N. Necker, David J. Cholok, Marc J. Fischer, Kyle Gifford, Chris Le Castillo, Michael Scholz, Michael Januszyk, Christoph W. Leuze, Bruce L. Daniel, Arash Momeni

**Affiliations:** ^1^ Digital Anatomy Lab Faculty of Medicine Institute of Functional and Clinical Anatomy Friedrich‐Alexander Universität Erlangen‐Nürnberg (FAU) Erlangen Germany; ^2^ Department of Radiology Stanford IMMERS (Incubator for Medical Mixed and Extended Reality at Stanford) Stanford University School of Medicine Palo Alto California USA; ^3^ Division of Plastic and Reconstructive Surgery Stanford University School of Medicine Palo Alto California USA; ^4^ Department of Radiology, 3D and Quantitative Imaging Stanford University School of Medicine Stanford California USA

**Keywords:** 3D‐printed DIEP‐flaps, accuracy validation, perforator location, perforator mapping, perspective‐corrected photographs, skin‐surface measurements

## Abstract

**Background:**

We present a novel method for accurately measuring skin‐surface distances using standard smartphone photos and Photoshop, validated on 3D‐printed DIEP‐flap models and on calibration grid‐patterns.

**Materials and Methods:**

Distance measurements are acquired in Photoshop in a calibration plane between dots on a grid‐pattern as well as between perforators on photos of 3D‐printed models and compared against ground‐truth. Margins of errors are calculated from fitted linear models.

**Results:**

Submillimeter accuracy can be achieved within errors of ±0.45 mm (80% probability) and ±0.8 mm (95% probability) for measuring distances on the dot‐grid. On the 3D‐printed DIEP‐models, distance measurements are accurate within ±1.75 mm (80% probability) and ±3.1 mm (95% probability).

**Conclusions:**

We introduce a simple yet highly accurate technique to measure skin‐surface distances using normal photos. Depending on the scenario, submillimeter or conservatively very low millimetre errors can be achieved, sufficiently accurate for clinical use, whilst maintaining topographic relationships of the measurements.

## 1 Introduction

Easy comparison of anatomical features across individuals and time‐points along the peri‐operative course is dependent on accurate measurement of distances between anatomic landmarks. These distance measurements are not only important to establish quantifiable objectivity of perceived features (e.g., in aesthetics [1]) but also to ensure accuracy, consistency, and standardisation of perioperative surgical outcomes [2, 3, 4, 5].

**FIGURE 1:**
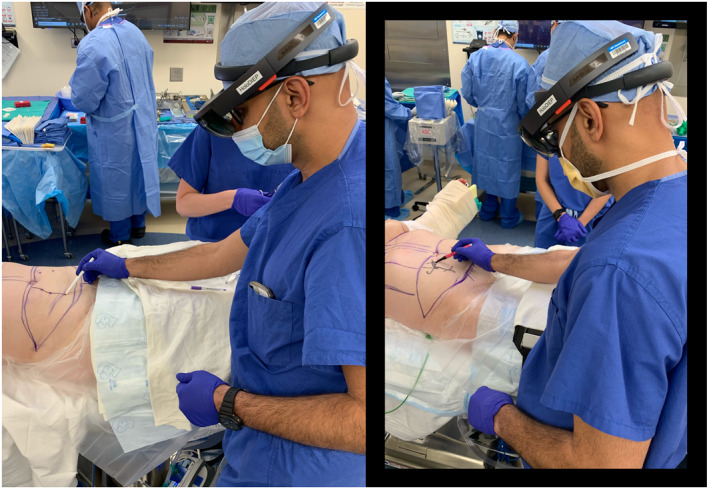
Surgeon using a new experimental software looking through HoloLens Mixed‐Reality glasses to trace the DIEA vascular tree and its perforators onto two patients' abdomens.

**FIGURE 2:**
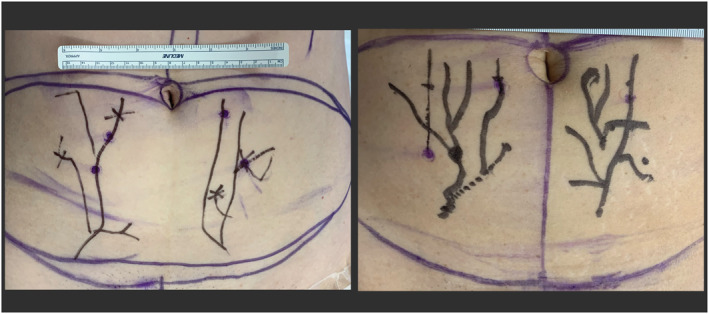
Two photos of experimental abdominal perforator maps created with an experimental Mixed‐Reality tool that could be analysed with this technique in the future.

New techniques for mapping perforator locations onto the abdomen allow surgeons to visualise perforators with ‘x‐ray vision’ using advanced projections and holograms [6, 7, 8, 9, 10, 11]. Perforators exiting the fascia can be located shining through translucent skin and consecutively be drawn directly onto the patient abdomen (Figure 1). As an exemplary application where multiple measurements need to be acquired at once, two perforator mappings created with an experimental Mixed‐Reality tool are shown in Figure 2, where the surgeon has drawn the vascular tree of the Deep Inferior Epigastric Artery onto patients' abdomens guided by a hologram.

Similarly to perioperative surgical outcomes, for validating the accuracy, consistency, and standardisation of such perforator mappings, distances between perforator locations on the skin could be compared against ground truth anatomy from radiological imaging, since CT angiography is routinely used for abdominal perforator flaps [12, 13, 14, 15, 16]. Unfortunately, current techniques of measuring skin‐surface distances in vivo are either inconsistent, prohibitively expensive, or impractical for intraoperative use. Plastic surgeons need a simple, fast and accurate measuring methodology to measure distances on skin‐surface level.

Preoperatively, skin‐surface distance measurements are commonly taken using a ruler or calliper on the patient's body surface [17, 18, 19, 20]. These anthropomorphic measurements in the context of soft‐tissue have been primarily studied on the breast, revealing partial inconsistencies in reliability attributed to inter‐rater reliability, soft‐tissue deformation and variable anatomical landmarks on the breast [21, 22]. The accuracy and reliability of ruler measurements on the abdomen has not been studied yet, due to missing clinical needs. We anticipate that ruler measurements on the abdomen might similarly be susceptible to soft‐tissue deformation as measurements on the breast, even though less significantly.

Increasingly, three‐dimensional photography is becoming the standard for highly‐detailed surface measurements, allowing assessment of multiple skin‐surface distances, especially in pre‐/post‐operative comparisons [23, 24, 25, 26]. Even though hand‐held photogrammetry devices are very accurate in the OR [27, 28], routine use is limited due to complexity, space constraints and unaffordability—sterile intraoperative use by surgeons is a novel rarity. Highly accurate stereotactic systems, already used in the fields of neurosurgery [29] and orthopaedic surgery [30, 31], are prohibitively expensive and otherwise not needed for guidance in plastic surgery. Therefore, plastic surgeons mostly resort back to the simplest measuring technique in the OR: the sterile tape ruler.

However, measuring with a ruler presents several disadvantages for intraoperative use. Measurements need to be recorded separately, which might lead to confusion when multiple similar measurements need to be acquired quickly. It is especially difficult to acquire multiple measurements based on a common origin without shifting the ruler. Accuracy of ruler measurements remains user‐dependent and is never fully objective or reproducible (e.g., due to tissue deforming when additional measurements need to be acquired post‐hoc).

We propose a simple workflow based on perspective‐correction of smartphone photos and ruler‐analogous measurement in common image‐editing software. With a single image taken intraoperatively, germane distances can later be measured on this single photo. To simulate measuring skin‐surface distances in vivo, we validated our technique in two different scenarios. First, under ideal conditions using calibrated grid‐patterns printed on paper. Secondly, in a real‐world scenario, measuring perforator distances on photos of 3D‐printed DIEP‐flaps against 3D‐model ground‐truth from CT angiography. The 3D‐prints representing true patient anatomy at the fascia‐level are thereby used as substitutes simulating skin‐surface markings from perforator mapping.

## 2 Materials and Methods

Ethics approval from our human subjects research board (Institutional Review Board, IRB) was obtained for retrospective analysis. All patient specific information was de‐identified consistent with a Waiver of Individual Authorisation under 45 CFR 164.512(i)(2)(ii)(A),(B),(C).

### 2.1 Model Creation and Selection

**FIGURE 3:**
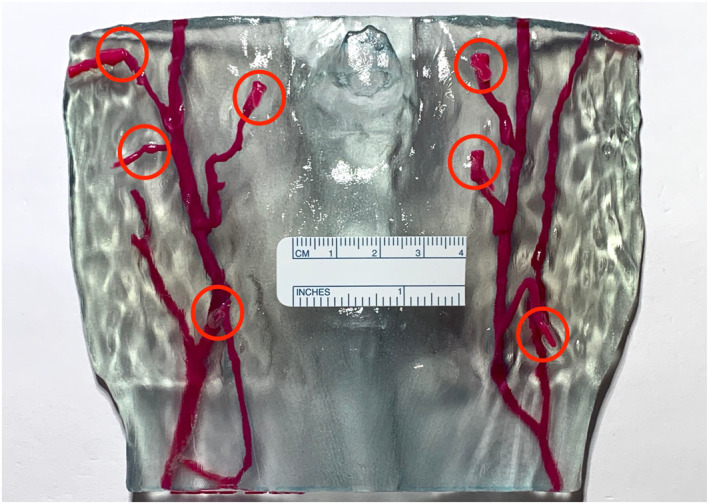
One of the 3D‐printed DIEP models with perforator locations exiting at the Rectus Abdominis Muscle highlighted with red circles.

A total of 21 de‐identified 3D‐printed abdominal models were consecutively selected from one reconstructive surgeon's practice. At our institution, these 3D‐models are routinely created for all abdominal free‐flap breast reconstructions with preoperative CT imaging from high‐resolution mixed arterial‐venous phase CT angiography as previously described [32]. The 3D‐printed models provide an accurate physical representation of the anatomic detail from high‐resolution radiological images featuring a translucent blue Rectus Abdominis Muscle and an opaque red Deep Inferior Epigastric Artery (DIEA) vascular tree [32]. DIEA perforators (DIEP) exiting the translucent muscle at the fascia level (referred to as perforator locations) are clearly differentiable (Figure 3).

An isometrically spaced dot‐grid pattern (10 mm) of 0.5‐point sized dots (vector graphics) is printed at high‐resolution (600dpi) on a laser printer (Utax 2508ci, TA Triumph Adler, Nürnberg, Germany). Different ground‐truth distances are inherent the grid‐pattern itself, for example the distance between the first and second dot is 1 cm, between first and third is 2 cm and so on. 10 pictures of the grid‐papers were taken using a telephoto lens (2X magnification) in a smartphone (iPhone XS (12th generation), Apple Computer, Cupertino, CA, USA). The images were taken perpendicular to the paper utilising the iPhone's built‐in levelling‐crosshair feature in the ‘Camera’ app from roughly 50 cm afar, a feasible and practical distance for sterile and non‐sterile intraoperative camera handling, to frame the entire paper sheet corresponding to a prototypical patient abdomen and surgical field. For the second scenario, images of the 3D‐printed models were taken with the same approach. Additionally, a ruler was positioned at the middle fascia‐surface level close to the umbilicus for calibration only (Figure 3).

**FIGURE 4:**
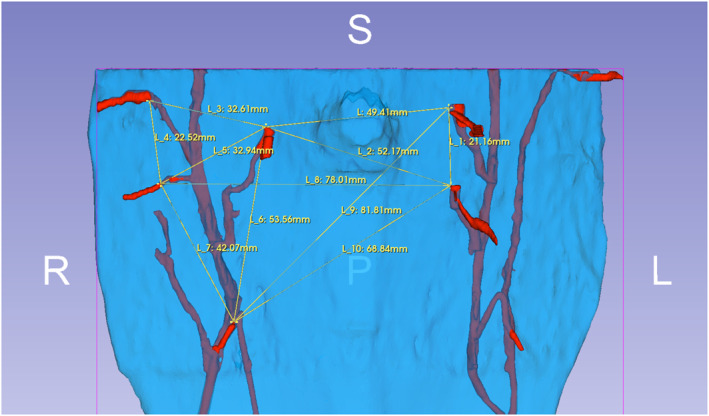
3D ground truth model visualised in 3D Slicer with distance measurements in yellow. Same patient case as in Figure 1. Note that the lower left perforator was not used for measurements due to overlapping on photographs not allowing an unambiguous point to be identified.

To establish ground‐truth measurements, 3D‐models were loaded into 3D Slicer [33, 34, 35], an open‐source radiology and medical imaging software, where inter‐perforator distances (yellow) were measured on the fascia‐surface for feasible combinations of perforators (Figure 4) and exported with a customised script [36] to an Excel spreadsheet (Microsoft, Redmond, WA, USA). We chose points that were clearly and unambiguously identifiable as a common reference on the 3D‐models and photos of the 3D‐prints alike.

To acquire the photo measurements, images were opened in Photoshop 2022 (Adobe Inc., San Jose, CA, USA) and distortion‐corrected using the ‘Lens correction’ filter with integrated presets (e.g., for our iPhone XS). Next, a measurement plane was calibrated to the known dimensions of the grid‐pattern or the surgical ruler using the ‘Vanishing Point’ plugin. In an ideal photo taken perfectly perpendicular to a surface, any length is directly proportional to the known dimensions of a calibration object. The ‘Vanishing Point’ filter corrects for the off‐axis perspective present in handheld photos and scales measurements accordingly. All measurements acquired with this technique are therefore considered planar distance measurements in the calibration plane, respectively, abdominal skin‐surface plane.

**FIGURE 5:**
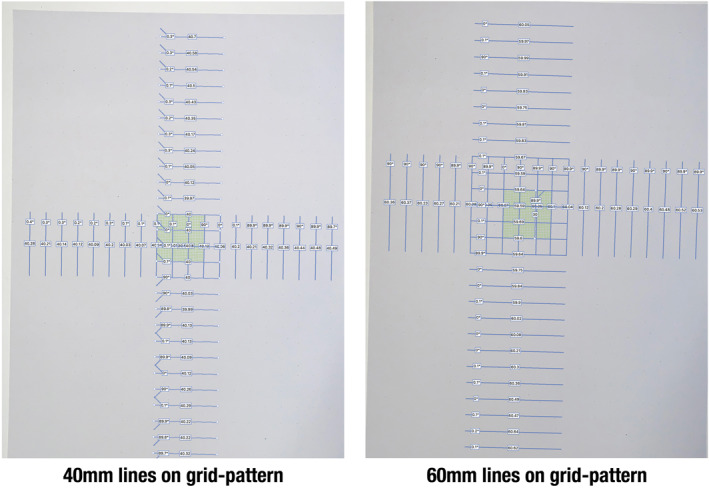
Screenshot from Photoshop demonstrating examples of 40 and 60 mm measurements systematically taken on photos of the grid‐pattern. Calibration plane rectangle in yellow‐green. Dot‐pattern barely visible.

For the grid measurements, 30, 40, 50, 60 and 70 mm distances inherent to the grid‐pattern were measured on the 10 different photos (Figure 5).

**FIGURE 6:**
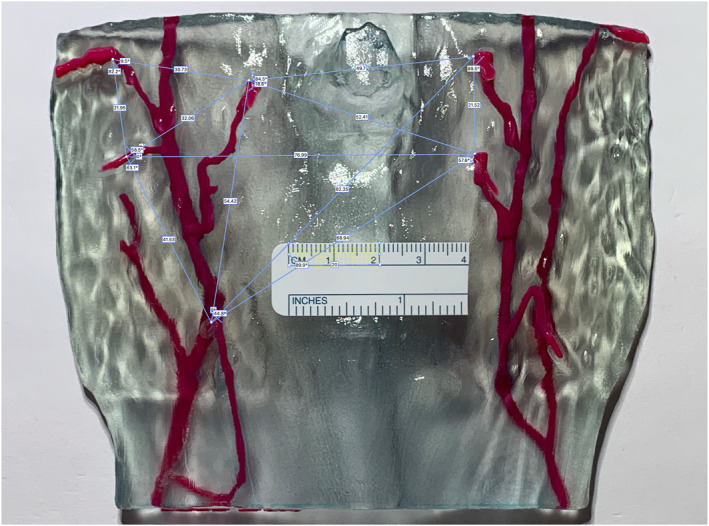
Screenshot from the measurements in Photoshop acquired on the image of 3D‐printed DIEP‐flap model—calibration plane rectangle in yellow. Note, that the same unambiguous points as previously defined on the 3D‐model in 3D Slicer (Figure 4) were used.

For the 3D‐model measurements, we used the surgical ruler to define the rectangular calibration object using 30 mm of the ruler's marking for scaling. Analogous to the measurements performed in 3D Slicer on the 3D‐model, the same distances were measured in the photos of the models and recorded (Figure 6).

### 2.2 Statistical Analysis

Statistical analysis was performed in RStudio [37] (version 2022.7.2.576) using the packages ‘mountainblot’, ‘blandr’, ‘rcompanion’, ‘scales’, ‘gamlss’, ‘WRS2’ for statistical analysis and ‘ggplot2’ for visualisation.

Distance differences between ground‐truth (Method 1) and photo measurements (Method 2, our method) acquired with our technique will be referred to as grid differences and model differences, respectively. We fitted appropriate error‐prediction functions on these distance differences with ‘fitDist’ from ‘gamlss’ (Generalised Additive Models for Location, Scale and Shape).

We approximated 80, 90, 95% quantiles with the empirical cumulative distribution function (ECDF) to investigate the margins of error for 80, 90, 95% of all measurements and compared these to our fitted models (inverse cumulative distribution function (ICDF)). The ECDF was plotted as mountainblots folded around the median, allowing easier visual comparison with the error‐prediction functions' fitted quantiles and the cumulative probabilities of absolute errors.

Barplots display cumulative probabilities for absolute errors, for example the probability to measure accurately within ±xmm, sampled from our fitted function after confirming their sufficient accuracy. Bland‐Altman method comparison was used to investigate bias between grid‐pattern or 3D‐model ground‐truth (Method 1) and their respective photo measurements (Method 2) on a 95% significance level. Additionally, proportional Bland‐Altman analysis visualises proportional error dependent on the magnitude of the distance to be measured.

## 3 Results

Setting the calibration plane normally takes around 2 min for grid‐ and 3D‐model measurement scenarios. Precision in this step was found to be crucial to minimise errors. Distance measurements themselves are then done within seconds. Measuring an entire abdominal 3D‐print with multiple perforators normally took less than 5 min start to finish.

**FIGURE 7:**
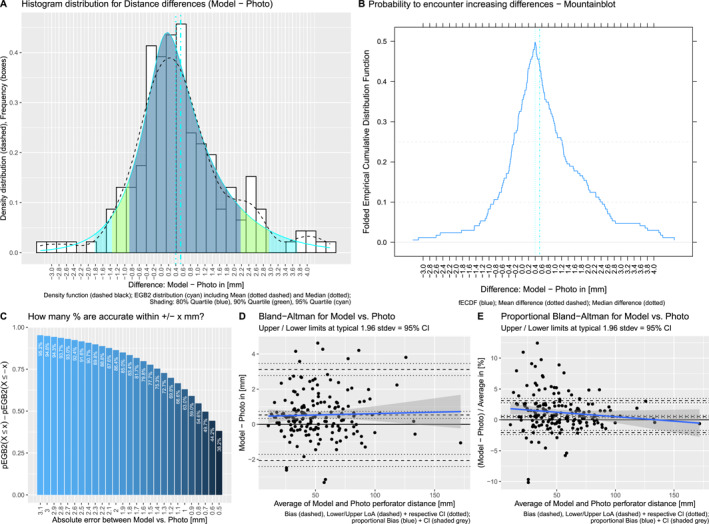
Analysis of the differences between grid and photo measurements. Histogram of the probability distribution (to encounter errors between our technique and the real‐world grid geometry) with density, fitted curves and shaded quantiles indicates a skewed‐normal distribution, where our technique is minimally overestimating (A). Mountain blot for the cumulative probabilities at increasing distances visualises more intuitively that 80% (mountain part above the 0.1) of measurements are within −0.6 and 0.3 mm error and 50% (mountain part above the dashed 0.25) are within −0.4 and 0.0 mm (B). Boxplot of combined probabilities at absolute distances makes this more intuitive as a plus‐minus‐range, that is, for the previous example, 80% falls within approx. ± 0.45 mm and 50% within approx. ± 0.2 mm (C). Bland‐Altman analysis (D) and proportional Bland‐Altman analysis (E) indicate a minimal bias in our technique overestimating increasingly long distances; however, proportional to the length itself, the error is considerably small.

**FIGURE 8:**
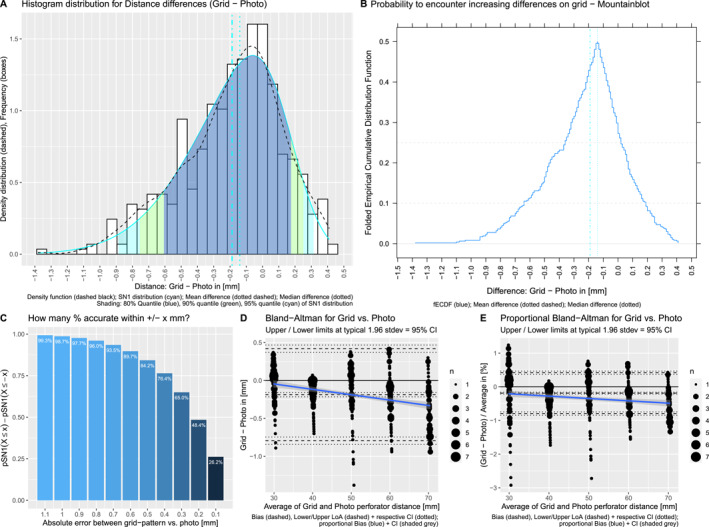
Following exactly the same structure as Figure 7, but this time with analysis on the differences between 3D‐DIEP‐models and photo measurements. Histogram shows the probability of measurement errors (meaning the difference between measurements on the model and photo) a somewhat normal distribution that is slightly left‐skewed indicating that our technique overestimates measurements minimally (A). Mountainblot for the cumulative probabilities at increasing distances indicating that for 80% (mountain‐part above 0.1) our technique is within −0.8–2.4 mm accurate (B). Boxplot of combined probabilities at absolute distances visualises the results from (B) more intuitively as plus‐minus‐ranges, for example for 80% of measurements we are within ± 1.6 mm accurate (C) with Bland‐Altman analysis (D) and proportional Bland‐Altman analysis demonstrate very limited bias in our technique, even in this analysis on the 3D models mimicking real‐world conditions (E).

For the grid measurements, 465 photo measurements were taken on 10 photos, 5 calibrated horizontally and 5 vertically. For the 3D‐model measurements, 21 patient cases yielded 168 perforator measurements with varying numbers per case. Shapiro‐Wilk test and QQ‐plots confirmed non‐normal distributions of differences for both scenarios. Significant skewedness in both difference distributions can be observed in the histograms (Figures 7 and 8).

We used Wilcoxon Robust Statistical Methods 2 package (WRS2) for descriptive statistics (10% trimmed mean), since trimmed means have a better central tendency for non‐normally distributed data. The grid differences are on average −0.169 mm (−0.2002 mm to −0.1375 mm 95% CI mean) and the model differences are on average 0.468 mm (0.2699–0.6662 mm 95% CI mean) (additional descriptive statistics in Supporting Information S1: Table S1).

Distribution fitting for the grid differences generated a Skew normal type 1 distribution (Azzalini type 1 maximising AIC = 198.9 and BIC = 211.3) (coefficients in Supporting Information S1: Table S2). Model‐differences were best fitted with an Exponential generalised beta 2 (EBG2) distribution (maximising AIC = 556.0 and BIC = 568.5) (coefficients in Supporting Information S1: Table S3).

The fitted functions are plotted as a cyan line on the histogram (empirical differences) with their 80, 90, 95% quantiles shaded in blue, green, cyan respectively (Figures 7 and 8). Each probability quantile represents the corresponding range of error for 80, 90, 95% of measurements. Fitted quantiles are compared with empirical quantiles at the closest captured values from the empirical cumulative distribution functions (Table 1).

**TABLE 1:** Comparison of fitted and empirical 80, 90, 95% quantiles for grids (column 2, 3) and models (column 4, 5) demonstrates very little deviation consecutively allowing acceptance of fitted functions for further predictions.

Quantile	Lower limit (grids) [mm]	Upper limit (grids) [mm]	Lower limit (models) [mm]	Upper limit (models) [mm]
80% empirical	−0.64	0.19	−0.8644	2.3764
80% fitted	−0.6063	0.1751	−0.874	2.1767
**80% deviation**	**−0.0337**	**0.0149**	**0.0096**	**0.1997**
90% empirical	−0.77	0.25	−1.264	2.7398
90% fitted	−0.7543	0.2511	−1.3483	2.9508
**90% deviation**	**−0.0157**	**−0.0011**	**0.0843**	**−0.211**
95% empirical	−0.89	0.34	−1.8384	3.8092
95% fitted	−0.8865	0.314	−1.8222	3.725
**95% deviation**	**−0.0035**	**0.026**	**−0.0162**	**0.0842**

*Note:* The bold values (“Deviation”) is the difference between the “Empirical” and “Fitted” values and are meant as an indicator for accuracy of our prediction model for probability estimation.

For 95% of grid measurements, the fitted functions and empirical observation deviate 0.03 mm at most. Therefore, we consider the fitted function sufficiently accurate for further predictions. Predicting from the function, 80% of grid photo measurements accurately measure within −0.61 to 0.18 mm, 90% within −0.75 to 0.25 mm and 95% within −0.89–0.31 mm of difference from ground truth (Figure 7).

For 95% of abdominal 3D‐model measurements, the fitted functions deviate 0.2 mm at most from empirical observation and are therefore sufficiently accurate for further predictions. 80% of 3D‐model photo measurements accurately measure within −0.87–2.18 mm, 90% within −1.35–2.95 mm and 95% within −1.84–3.73 mm difference from ground truth (Figure 8).

The mountainplots folded at 50% (Figures 7 and 8) give a more intuitive understanding of these quantiles on the empirical measurement differences: the mountain above a certain probability threshold captures the accepted range of errors in distance. We can visually observe that 80% of differences (above the 10%/90% horizontal threshold) lie within −0.65 to 0.2 mm for the grids and within −0.9–2.4 mm for the model, visually confirming the functions' fit described previously.

However, a more practical consideration is to think about the probability for accurate measurement within an absolute margin of error of $\pm \;x_{i}\;\text{mm}$ from ground‐truth. Instead of using standard deviations and the mean due to skewedness, we calculate $P\left(X\leq x_{i}\right)-P\left(X\leq -x_{i}\right)$ representing the cumulative probability for errors between $\pm {\;x}_{i}\;\text{mm}$. The combined probabilities are shown in bar charts (Figures 7 and 8).

For the grids, approximately 80% of measurements will be within errors of ±0.45mm, 90% within ±0.6mm and 95% within ±0.8mm. This demonstrates submillimeter errors in both directions with high confidence.

For the models, approximately 80% of measurements will be within errors of ±1.75mm, 90% within ±2.4mm and 95% within ±3.1mm.

Intuitively, these plots also show that our photo measurement technique performs quite consistently for accepted errors of up to ±0.6mm for the grids and ±2.0mm for the models and then starts to deteriorate for smaller accepted errors.

Lastly, we perform Bland‐Altman methods comparison (using the blandr package) to investigate structural bias at typical limits of agreements set at 1.96 standard deviations (corresponding to a 95% significance level). Despite non‐normality, we used standard limits of agreements (LOA) for better comparability since most captured values are well within the LOAs. Additionally, proportional Bland‐Altman comparisons with the same parameters are shown to visualise percentual changes depending on the magnitude of the distance measured. Our photo technique used on the grids has an average overestimating bias of −0.187 mm (CI: −0.216 mm to −0.159 mm) and used on the models has a structural underestimating bias of 0.533 mm (CI: 0.332–0.734 mm).

For the grid measurements, Bland‐Altman reveals that the photo technique is only slightly overestimating for shorter and increasingly overestimating for longer distances but stays well within the limits of agreements for the majority of measurements. Notably, in the proportional Bland‐Altman it becomes apparent that the technique counterintuitively works most accurately for small distance measurements becoming less accurate with larger distances (blue downward trend not crossing zero, Figure 7), always remaining within 0.5% bias.

For the 3D‐model measurements, Bland‐Altman shows a relatively consistent absolute underestimating bias of our photo technique, regardless of the magnitude of the measured distance (Figure 8). The confidence interval of the bias continually increasing can be explained by a decreasing number of samples for big measurements and some obvious outliers. In contrast to the grids, in the proportional plot there is a downwards trend for the proportional bias (blue line getting close to zero)—for larger measurements, smaller absolute errors stemming from bias become less relevant.

## 4 Discussion

In this preclinical validation study, we validated a technique that enables accurate measurement of skin‐surface distances on the abdomen from a single smartphone photo to allow for easy, standardized analysis of perforator mappings. As it does not require any additional equipment and minimal learning curve, it could easily be implemented by other surgeons using any camera and a sterile object of known dimensions to assess multiple surface distances at once with high accuracy.

Under ideal conditions (grids) 80% of measurements acquired with the technique are accurate within ±0.45mm error and 95% within ±0.8mm. Under real‐world conditions (3D‐models) 80% of measurements are accurate within ±1.75mm, 95% within ±3.1mm error. Assuming ideal conditions, submillimeter accuracy is challenging expensive high‐end techniques like three‐dimensional photography.

Being able to correlate measurement and anatomical landmark (e.g., perforator location) anytime on the photo is a significant advantage over simple ruler measurement that cannot capture these topographical relationships. As abdominal soft tissue will remain susceptible to deformation, even just from pressure when performing a ruler measurement itself, this will never allow perfect measurements. Considering this fact, the biases and inaccuracies in our technique conclusively would be acceptable and sufficient for an affordable, quick technique which would not significantly interrupt surgical flow and could be analysed post‐operatively in a standardized manner.

We believe this technique could be used in the context of a variety of settings and procedures. To further validate this technique, we propose a step‐by‐step approach towards gradual clinical validation.

First, additional pre‐clinical studies would be needed to strengthen the accuracy analysis across a variety of different cases, procedures and settings in a theoretical lab setup. For example, study designs similar to ours could be performed on 3D‐printed models for another type of surgery, where 3D‐prints are routinely used. For example, craniomaxillofacial surgery of the mandible and jaw has fully established 3D‐printed guides and the rigid bony anatomy allows unequivocal identification of anatomical landmarks used as measurement targets.

Second, a logical first clinical step would be to perform in vivo anthropomorphic measurements on patients in an outpatient setting side‐by‐side with existing techniques. As described in the beginning, particularly in plastic surgery, a variety of measurements are taken during the pre‐operative planning phase during outpatient consultation. Often, these on‐skin distance measurements are performed in a standardized system, where besides the currently used standard measurement technique (mostly rulers/callipers) our technique could be used concurrently and then compared directly against reference techniques. For example, we believe our technique could be valuable for planning blepharoplastic and ophthalmoplastic surgeries because the distances around the eye and orbita are relatively small, thus benefiting from more accurate measurement, especially with the increased accuracy of our technique for measuring such small distances. Periorbital landmarks are inherently difficult to measure with a ruler and calliper (e.g., due to movement, blinking and patient discomfort) and additionally, the anatomy of the periorbita and midface is relatively flat, thus not being especially susceptible to errors from curvature. Additionally, blepharoplasty is a high‐volume procedure that increasingly needs patient‐specific preoperative planning and thus would allow to quickly validate the technique across a large patient cohort, whilst providing a significant clinical benefit enabling easier planning measurements.

Third, validation of guidance accuracy in clinical perforator mapping scenarios starting with the DIEP‐flap. Following potential first measurements with our technique during preoperative outpatient consultations, a second set of measurements could be acquired in the OR prior to the first incision to validate how accurately these measurements remain in different real‐world settings of the same patient, including typical confounders such as tissue deformation. Given sufficient temporal stability and sufficiently high accuracy in real‐world settings, the use‐case could be expanded to intraoperative measurements during dissection, thus far not being explored in the literature.

Fourth, expanding to closely related yet distinct procedures could be considered, for example the Anterolateral Thigh Flap (ALT)—a general ‘workhorse’ reconstruction flap used in a variety of cases ‐ where the perforators originating in the lateral femoral circumflex artery are used. These perforators are even more variable and patient‐specific, thus warranting further studies into their anatomical variability and viability, as current studies are limited. Similar to studying perforators on the abdomen, in this case the location of the lateral thigh perforators to surrounding anatomical landmarks (i.e., the anterior superior iliac spine) could be studied under similar conditions in perforator mapping studies.

One potential explanation for the different outcomes between the two scenarios in our study could be topography: whilst the grids were perfectly flat, the digital 3D‐models were obviously not flat since true surface distances were measured as one would do with a real tape measure. In the photo measurements, one cannot account for curvature from depth as all points are assumed to be in the same plane. Anecdotally, we observed that for points closer to the lateral edges of the rectus muscle and therefore on the most steeply curved parts, differences would be bigger.

In our case of using this technique for analysing DIEA perforators, the most relevant perforators were located relatively close to the umbilicus and not on the far‐lateral edges of the abdomen [38, 39]. Whereas perforator locations here were measured on the quite steeply curved fascia‐level, in a supine intraoperative position, even more voluminous abdomens feature relatively flat topography around the umbilicus at the skin‐surface level, the region of interest when perforator mapping. Therefore, even in these circumstances, our technique should perform well for its intended purpose. Additionally, curvature becomes less relevant as measurement length increases, as demonstrated by the decreasing proportional bias, which would be the case for perforators located at the lateral sides of the abdomen.

A potential limitation in general might be the time required for performing calibration and measurements in the operating room, which we estimate to be around 2 min. In certain high‐pressure scenarios, where interruption is unwarranted, our technique might not be sufficiently fast. However, as soon as multiple measurements need to be acquired at once, we believe our technique can be much faster than the conventional sterile ruler/tape measure technique, whilst simultaneously allowing to investigate multiple spatial relationships at once, even with additional time needed for post‐processing.

A limitation of our model study was the limited number of measurements due to limited case number—including more measurements, errors would further decrease as outliers become less significant. Another limitation is the sensitivity of the calibration rectangle—changes to the geometry could partially impact measurements if calibrated carelessly. Lastly, considering the added time needed, this technique primarily provides benefit over normal ruler measurements when multiple measurements need to be performed in a standardized manner whilst maintaining record of their topographic relationships and when high accuracy for very small measurements is needed, for example when comparing perforator mappings.

**FIGURE 9:**
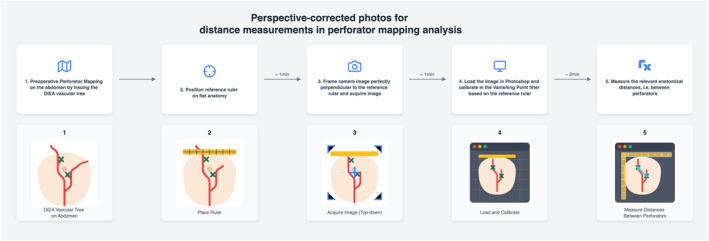
This figure exemplifies step‐by‐step how our technique can be used for analysing DIEA perforator mappings and could be clinically integrated.

Future studies are needed to investigate additional sources of inaccuracies that might influence clinical reliability (e.g., skin types) as well as comparative analysis against established reference measurement techniques for measurements and procedures where such reference techniques are established. We envision this technique to be clinically integrated and used as a part of a comprehensive workflow to analyse the accuracy and consistency of perforator mapping in reconstructive surgery (Figure 9).

Intraoperative 2D photography is already commonly used [40] for documentation, communication and teaching in plastic surgery and can easily be augmented using this technique. Looking to the future, this technique could also be implemented with other smaller sterile objects instead of the ruler. Any rectangular, sterile tool with known dimensions would work. Photos could be acquired by either a non‐scrubbed team member with any existing camera, by the increasingly common cameras integrated in surgical lights, or by scrubbed surgeons themselves with affordable sterile smartphone covers. It can also be used to postoperatively measure photos that were already acquired as per standard surgical workflow.

## 5 Conclusion

We introduce a new technique for skin‐surface distance measurements on perspective‐corrected photographs that can be acquired with any camera.

Eighty percent of measurements can be measured with submillimeter accuracy within ±0.45mm under ideal condition and within ±1.75mm under real‐world conditions evaluated on 3D‐printed DIEP‐flaps warranting future use for analysing perforator mapping accuracy.

## Author Contributions

**Fabian N. Necker:** conceptualization, data curation, formal analysis, project administration, investigation, validation, writing – original draft preparation, writing – review and editing. **David J. Cholok:** investigation, writing – original draft preparation, writing – review and editing. **Marc J. Fischer:** formal analysis, software, writing – review and editing. **Kyle Gifford:** data curation, investigation, writing – review and editing. **Chris Le Castillo:** data curation, investigation, writing – review and editing. **Michael Scholz:** funding acquisition, writing – review and editing, supervision. **Michael Januszyk:** formal analysis, methodology, validation, writing – review and editing. **Christoph W. Leuze:** conceptualisation, methodology, writing – review and editing. **Bruce L. Daniel:** conceptualisation, writing – review and editing, supervision, project administration, resources. **Arash Momeni:** conceptualization, writing – original draft preparation, writing – review and editing, supervision, project administration.

## Conflicts of Interest

F. Necker is a part‐time research student at Siemens Healthineers (Erlangen, Germany). Dr. C.W. Leuze is a co‐founder and owner of Nakamir Inc (Menlo Park, CA). Dr. A. Momeni is a consultant for AxoGen, Gore, RTI, and Sientra. The remaining authors have no conflict of interest to disclose.

## Permission to Reproduce Material From Other Sources

The authors have nothing to report.

## Supporting information


Supporting Information S1

## Data Availability

Data can be requested from the corresponding author upon reasonable request.
