# Supplementary material for: Measuring Abdominal Skin‐Surface Distances Using Photos for Perforator Mapping Analysis—A Validation Study on 3D‐Printed DIEP‐Flap Models
Source: Int J Med Robot. 2025 Sep 17;21(5):e70108. doi: 10.1002/rcs.70108 (PMC12444031; doi:10.1002/rcs.70108)
Supplement: Supplementary file 1 — Supporting Information S1 [file RCS-21-e70108-s001.docx]

Table S1: extended descriptive statistics on the grid and model differences.

| Min. | 1st Qu. | Median | **Lower 95% CI on 10% trimmed mean** | **10% trimmed mean** | **Upper 95% CI on 10% trimmed mean** | **SE of 10% trimmed mean** | Mean | 3rd Qu. | Max. |  |
| --- | --- | --- | --- | --- | --- | --- | --- | --- | --- | --- |
| Grids | -1.38 | -0.36 | -0.14 | **-0.2002** | **-0.1689** | **-0.1375** | **0.0151** | -0.1874 | 0.02 | 0.41 |
| Model | -3.3092 | -0.2558 | 0.3975 | **0.2699** | **0.468** | **0.6662** | **0.0837** | 0.5332 | 1.1902 | 4.6216 |

Table S2: distribution fitting coefficients for a Skew normal type 1 function modelling the grid differences.

|  | **Estimate** | **Standard Error** | **t value** | **Pr(>\|t\|)** |
| --- | --- | --- | --- | --- |
| eta.mu | 0.1662573 | 0.0228314 | 7.28195 | 3.29e-13 |
| eta.sigma | -0.7556806 | 0.0491414 | -15.37769 | < 2.22e-16 |
| eta.nu | -2.853188 | 0.4521135 | -6.31078 | 2.78e-10 |

Table S3: distribution fitting coefficients for an Exponential generalized Beta 2 function modelling the Model differences.

|  | **Estimate** | **Std. Error** | **t value** | **Pr(>\|t\|)** |
| --- | --- | --- | --- | --- |
| eta.mu | 0.0760617 | 0.147471 | 0.51577 | 0.606012 |
| eta.sigma | -1.6912491 | 0.7348753 | -2.30141 | 0.021368 |
| eta.nu | -1.3110318 | 0.8138932 | -1.61082 | 0.10722 |
| eta.tau | -1.8017705 | 0.7863083 | -2.29143 | 0.021939 |
